# A Comparative Observational Study on the WHO Labour Care Guide Versus the WHO Modified Partograph on Labour Outcome

**DOI:** 10.7759/cureus.102080

**Published:** 2026-01-22

**Authors:** Kurukuntla Vishnu Priya, Shreedevi Kori, Rajasri G Yaliwal, Mudanur SR, Preeti Malapure, Laxmi Sangolli

**Affiliations:** 1 Obstetrics and Gynaecology, Shri B. M. Patil Medical College, Hospital and Research Centre, BLDE (Deemed to be University), Vijayapura, IND

**Keywords:** cesarean section, labour monitoring, obstetric care, who labour care guide, who modified partograph

## Abstract

Introduction: Labour is a critical physiological process requiring close monitoring to ensure optimal maternal and neonatal outcomes. Traditional labour monitoring using the WHO Modified Partograph has been widely adopted; however, concerns regarding rigid time thresholds and unnecessary interventions have prompted the development of the WHO Labour Care Guide (LCG), a more individualized and woman-centred tool aligned with contemporary evidence. Comparative data between these two tools remain limited. The study aimed to compare the effectiveness of the WHO LCG with the WHO Modified Partograph in terms of maternal labour outcomes and caesarean section rates among primigravida women at term.

Methods: This comparative observational study was conducted at a tertiary care teaching hospital in South India. A total of 146 primigravida women in spontaneous labour with singleton, cephalic pregnancies at 37-40 weeks of gestation were enrolled. Participants were equally allocated to monitoring with either the WHO LCG (n = 73) or the WHO Modified Partograph (n = 73). Maternal labour parameters, mode of delivery, hospital stay, and neonatal outcomes were recorded.

Results: Women monitored using the WHO LCG had significantly shorter durations of the active phase and the second stage of labour and a markedly lower caesarean section rate compared with those monitored using the Modified Partograph (p < 0.001). Vaginal delivery rates were significantly higher, and hospital stays were shorter in the LCG group. Neonatal outcomes, including APGAR (appearance, pulse, grimace, activity, respiration) scores, birth weight, NICU admissions, and NICU stay duration, were comparable between the two groups.

Conclusion: The WHO LCG is a more effective intrapartum monitoring tool than the WHO Modified Partograph, improving maternal labour outcomes without compromising neonatal safety. Its adoption may enhance labour efficiency, reduce unnecessary caesarean deliveries, and support woman-centred obstetric care.

## Introduction

Labour is the most crucial part of every woman’s life, requiring a coordinated interaction between effective uterine contractions, adequate maternal effort, favourable foetal characteristics, and appropriate pelvic anatomy [1]. A successful labour outcome is necessary to ensure maternal and neonatal well-being. However, abnormalities in labour progress can lead to serious complications, making close monitoring and skilled intrapartum management indispensable [2].

The active management of labour has remained a topic of considerable debate since the 1970s, attracting sustained discussion and controversy [1]. Obstetric practices vary widely despite the persistent burden of high maternal mortality in developing countries and a rising caesarean section rate in developed nations [3]. Notably, the increasing rate of operative deliveries has not consistently translated into improved foetal outcomes, highlighting the need for evidence-based labour monitoring strategies that promote safe vaginal delivery while avoiding unnecessary interventions [1,4].

Worldwide, maternal mortality has shown a substantial downward trend over the past two decades, with the maternal mortality ratio falling by approximately one-third, from 339 deaths per 100,000 live births in 2000 to 223 per 100,000 live births in 2020 [5]. Despite this encouraging decline, progress remains insufficient to meet the Sustainable Development Goal (SDG) 3 target of reducing maternal mortality to below 70 per 100,000 live births by 2030, particularly as low- and middle-income countries continue to account for nearly 95% of all maternal deaths globally [6]. In Uganda, notable improvements were observed between 2016 and 2022, during which maternal mortality decreased from 336 to 189 per 100,000 live births, alongside a reduction in infant mortality from 43 to 34 per 1,000 live births [7,8]. Nevertheless, obstructed labour persists as a significant public health challenge in resource-limited settings, contributing to more than 8% of maternal deaths and approximately 90% of perinatal deaths associated with birth asphyxia [6-9].

In India, despite ongoing efforts to improve obstetric outcomes, life-threatening complications during labour remain common. Obstetric haemorrhage in the immediate postpartum period is a main cause of mortality, while obstructed labour and uterine rupture in neglected cases account for more than two-thirds of maternal deaths [1]. In this context, skilled labour management using a partograph, a simple graphical tool for monitoring labour progress and maternal-foetal condition, plays a pivotal role in the early identification and prevention of prolonged labour and its complications [1]. The composite World Health Organization (WHO) partograph, introduced in 1994, included both latent and active phases of labour with alerts and action lines. However, concerns regarding inappropriate interventions during the latent phase led to the introduction of the modified WHO partograph in 2000, which excluded the latent phase to simplify use and improve clinical decision-making [10,11].

In 2018, the WHO released new recommendations aimed at promoting a better childbirth experience, redefining normal labour duration and emphasizing respectful maternity care [1,12,13]. To facilitate implementation of these recommendations, the WHO introduced the Labour Care Guide (LCG) in 2020. The LCG aligns labour monitoring with contemporary evidence on labour progression, intervention thresholds, and woman-centred care [13]. Although several studies have evaluated the WHO LCG and the modified WHO partograph individually, there remains a paucity of comparative studies assessing their relative effectiveness. Therefore, this study aimed to compare maternal and neonatal outcomes between women monitored using the WHO LCG and those monitored using the WHO Modified Partograph, focusing on labour duration, mode of delivery, and neonatal well-being.

## Materials and methods

This comparative observational study was conducted in the Department of Obstetrics and Gynaecology at Shri B.M. Patil Medical College Hospital and Research Centre, Vijayapura, Karnataka, India, from March 2024 to August 2025. All eligible primigravida women who presented to the labour room for delivery during the study period were included in the study. Institutional Ethics Committee approval was obtained prior to study initiation (BLDE(DU)/IEC/SBMPMC/066/2023-24).

All primigravida women with a gestational age between 37 and 40 weeks, in spontaneous labour with a singleton foetus in cephalic presentation, and without associated medical or obstetric comorbidities were included. Women were excluded if they underwent a caesarean section during the latent phase of labour or had a history of preeclampsia or gestational hypertension, gestational diabetes mellitus, or breech presentation.

The variables recorded included maternal age, literacy status, body mass index, gestational age at delivery, cervical dilatation at admission, duration of the active phase and second stage of labour, need for augmentation, mode of delivery, indications for caesarean section, occurrence of postpartum haemorrhage, and duration of hospital stay. Neonatal assessment included APGAR (appearance, pulse, grimace, activity, respiration) scores at one and five minutes after birth. An APGAR score of ≥8 was considered indicative of satisfactory neonatal adaptation, as per the standard APGAR scoring system originally described by APGAR and widely adopted in clinical practice [14]. Birth weight was recorded immediately after delivery, and normal birth weight was defined according to the WHO international standards [15]. Additional neonatal variables included NICU admission, indication for NICU admission, and duration of NICU stay.

Sample size estimation was performed using G*Power software version 3.1.9.4 (Heinrich Heine University Düsseldorf, Düsseldorf, Germany) [16]. The calculation was based on a comparison of proportions between two independent groups using a z-test for the difference between proportions (test family: Z tests; statistical test: proportions: two independent groups; type of power analysis: a priori). The expected effect size was derived from previously reported oxytocin augmentation rates (18.4% vs. 51.8%) by Pandey et al. [17]. A two-tailed significance level (α) of 0.01 and a statistical power (1 − β) of 96% were applied, resulting in a total sample size of 146 participants, with 73 women allocated to each group.

Labour monitoring in both groups followed standard institutional protocols. The Modified WHO Partograph defined the active phase of labour at 4 cm cervical dilatation and utilized fixed alert and action lines, without documenting supportive care interventions or mandating specific responses to deviations in labour parameters [10,11]. In contrast, the WHO LCG defined the active phase at 5 cm cervical dilatation, incorporated evidence-based time limits for labour progression, and emphasized intensified monitoring during the second stage of labour [12,13].

To minimize selection bias, all eligible women meeting the inclusion criteria during the study period were consecutively enrolled. Observer bias was minimized as the study team did not influence clinical decision-making and only extracted data from patient medical records.

Data were entered into Microsoft Excel (Microsoft Corporation, Redmond, Washington) and analyzed using IBM SPSS Statistics for Windows, Version 26 (Released 2019; IBM Corp., Armonk, New York). Continuous variables were expressed as mean and standard deviation and compared using the independent t-test, while categorical variables were expressed as frequencies and percentages and compared using the chi-square test. A p-value < 0.05 was considered statistically significant.

## Results

Baseline variables of participants were similar between cases and controls. The majority of women in both groups were ≤25 years of age (82.2% in cases (60) vs. 89.0% in controls (65)), with no significant difference in mean age. Literacy status was similarly distributed, with nearly equal proportions across all education categories. Nutritional profiles, assessed using BMI, showed that most participants were within the normal range, with no significant differences between groups. Overall, none of the baseline variables demonstrated statistically significant differences, confirming that the two groups were well matched at enrolment (Table 1).

**Table 1:** Baseline characteristics of study participants (cases vs. controls) Data are presented as frequencies and percentages or as mean ± standard deviation (SD). Comparison between cases and controls was performed using the chi-square test for categorical variables. A p-value < 0.05 was considered statistically significant. Abbreviations: BMI – Body Mass Index; SD – Standard Deviation.

Variable		Cases (n = 73)	Controls (n = 73)	Chi-square value	p-value
Age	≤ 25 years	60 (82.2%)	65 (89.0%)	0.56	0.453
	> 25 years	13 (17.8%)	8 (11.0%)		
	Mean ± SD	22.4 ± 0.39	22.0 ± 0.35		
Literacy status	Illiterate	15 (20.5%)	13 (17.8%)	1.15	0.916
	Primary	21 (28.8%)	23 (31.5%)		
	Secondary	19 (26.0%)	21 (28.8%)		
	Graduate	17 (23.3%)	14 (19.2%)		
	Post-graduate	1 (1.4%)	2 (2.7%)		
BMI (kg/m²)	Underweight (<18.5)	10 (13.7%)	16 (21.9%)	3.47	0.322
	Normal (18.5–22.9)	40 (54.8%)	42 (57.5%)		
	Overweight (23–24.9)	13 (17.8%)	10 (13.7%)		
	Obese (>25)	10 (13.7%)	5 (6.8%)		
	Mean ± SD	21.3 ± 1.4	20.7 ± 1.6		

Labour outcomes differed significantly between cases and controls across multiple parameters. All women in both groups required labour augmentation with oxytocin, with no significant difference observed (p = 1.00). Mean gestational age at delivery was also nearly identical. Cervical dilatation at the start of the partograph was significantly higher in cases (5.0 ± 0.0 cm) compared to controls (4.0 ± 0.0 cm) (p < 0.001). The duration of the active phase of labour was significantly shorter in cases (3.60 ± 0.50 hours) than in controls (4.13 ± 0.096 hours) (p < 0.001). In contrast, the second stage of labour was significantly longer among cases (15.84 ± 0.61 minutes) compared with controls (11.89 ± 0.81 minutes) (p < 0.001). Vaginal delivery was significantly more frequent in cases (70, 95.9%), whereas caesarean delivery was higher in controls (27.4% vs. 4.1%) (p < 0.001). Among caesarean deliveries, foetal distress was the most common cause in cases, while a prolonged first stage of labour predominated in controls, showing statistical significance (p = 0.01). Duration of hospital stay was significantly shorter among cases (23.19 ± 1.04 hours) compared to controls (29.90 ± 1.57 hours) (p < 0.001). The incidence of atonic postpartum haemorrhage was low and comparable between the groups (p = 0.64) (Table 2).

**Table 2:** Labour outcomes among cases and controls Data are expressed as frequencies and percentages or as means ± standard deviation (SD). Categorical variables were analyzed using the chi-square test, while continuous variables were compared using the independent Student’s t-test. A p-value < 0.05 was considered statistically significant. Abbreviations: LSCS – Lower Segment Caesarean Section; PPH – Postpartum Haemorrhage; SD – Standard Deviation.

Outcome		Cases (n = 73)	Controls (n = 73)	Test statistic	p-value
Gestational age (days)	Mean ± SD	309.18 ± 0.804	309.21 ± 0.849	t = 0.02	0.981
Need for augmentation (oxytocin)	5 Units	73 (100%)	73 (100%)	χ² = 0.00	1.00
Cervical dilation at the start of the partograph	Mean ± SD	5 ± 0.00	4 ± 0.00	t = 18.6	<0.001
Duration of active phase (hours)	Mean ± SD	3.60 ± 0.50	4.13 ± 0.096	t = 8.21	<0.001
Second stage (minutes)	Mean ± SD	15.84 ± 0.61	11.89 ± 0.81	t = 31.4	<0.001
Mode of delivery	Vaginal	70 (95.9%)	53 (72.6%)	χ² = 14.30	<0.001
	Caesarean	3 (4.1%)	20 (27.4%)		
Indications of LSCS	Foetal distress	2 (66.7%)	3 (15%)	χ² = 0.21	0.65
	Prolonged 1^st^ stage	1 (33.3%)	13 (65%)	χ² = 6.52	0.01
	Prolonged 2^nd^ stage	0 (0.0%)	4 (20%)	χ² = 2.41	0.12
Duration of hospital stay (hours)	Mean ± SD	23.19 ± 1.04	29.90 ± 1.57	t = 29.6	<0.001
Atonic PPH	N (%)	3 (4.1%)	2 (2.7%)	χ² = 0.22	0.64

Neonatal outcomes were comparable between cases and controls, with no statistically significant differences observed. APGAR scores ≥8 at one minute were recorded in 68 (93.2%) of cases and 66 (90.4%) of controls (p = 0.546), while at five minutes, scores ≥8 were observed in 71 (97.3%) and 70 (95.9%) of neonates, respectively (p = 0.649). Mean birth weight was similar between the groups (2.728 ± 0.254 kg in cases vs. 2.712 ± 0.449 kg in controls; p = 0.542). NICU admissions were infrequent and evenly distributed, with meconium-stained liquor occurring in two (2.7%) of neonates in both groups (p = 1.00) and respiratory distress in 1.4% of cases and two (2.7%) of controls (p = 0.56). The mean duration of NICU stay did not differ significantly between cases (2.1 ± 0.9 days) and controls (2.4 ± 1.1 days) (p = 0.488), indicating comparable neonatal outcomes in both groups (Table 3).

**Table 3:** Neonatal outcomes Data are presented as frequencies and percentages or as means ± standard deviation (SD). The chi-square test was used for comparison of categorical variables, and the independent Student’s t-test for continuous variables. A p-value < 0.05 was considered statistically significant. Abbreviations: APGAR – Appearance, Pulse, Grimace, Activity, Respiration; NICU – Neonatal Intensive Care Unit; SD – Standard Deviation.

Neonatal parameter		Cases (n = 73)	Controls (n = 73)	Test statistic	p-value
APGAR score at 1 min	≥8	68 (93.2%)	66 (90.4%)	χ² = 0.36	0.546
	<8	5 (6.8%)	7 (9.6%)		
APGAR score at 5 mins	≥8	71 (97.3%)	70 (95.9%)	χ² = 0.21	0.649
	<8	2 (2.7%)	3 (4.1%)		
Birth weight (kg)	Mean ± SD	2.728 ± 0.254	2.712 ± 0.449	t = 0.61	0.542
NICU admission	Meconium-stained liquor	2 (2.7%)	2 (2.7%)	χ² = 0.00	1.00
	Respiratory distress	1 (1.4%)	2 (2.7%)	χ² = 0.34	0.56
NICU stay (days)	Mean ± SD	2.1 ± 0.9	2.4 ± 1.1	t = 0.70	0.488

## Discussion

Baseline variables were similar, therefore minimizing confounding factors and enhancing the internal validity of the findings. A similar baseline equivalence was reported by Jogi et al. [18], who demonstrated uniformity across age, educational status, BMI, gravida status, gestational age, and laboratory parameters, allowing outcome differences to be primarily due to the monitoring tool rather than patient characteristics.

A significantly higher vaginal delivery rate and a markedly lower caesarean section rate in the LCG group were observed in the present study. Caesarean delivery occurred in only 4.1% of women monitored with the LCG compared with 27.4% in the Modified Partograph group. Ninama et al. [19] reported comparable findings, with normal vaginal delivery rates of 73.3% in the partograph-monitored group versus 57.3% in controls, attributing the reduction in operative deliveries to early detection of labour abnormalities. Pandey et al. [17] further demonstrated that LCG use reduced emergency caesarean sections by promoting individualized labour assessment rather than fixed time thresholds, while Mugyenyi et al. [20] reported a decline in emergency caesarean deliveries in rural settings following LCG implementation. These findings collectively suggest that the LCG facilitates physiological labour progression and reduces unnecessary surgical intervention.

Neonatal outcomes in the present study were reassuring and comparable between the two groups. Although all neonates in the LCG group had APGAR scores of 8-9, a small proportion (4.1%) in the Modified Partograph group had lower scores; however, this difference was not statistically significant. Singh et al. [21] similarly observed no significant difference in APGAR scores, despite marginally higher mean scores in the LCG cohort. Jogi et al. [18] also reported slightly improved APGAR scores in the LCG group, while Srividya et al. [1] found no distinct neonatal advantage with LCG use. These findings indicate that while LCG may contribute to smoother labour progression, neonatal condition at birth remains comparable when appropriate intrapartum care is provided.

With respect to maternal labour dynamics, the present study demonstrated significantly shorter durations of the active phase and second stage of labour among women monitored with the LCG, accompanied by a reduced hospital stay. Jogi et al. [18] reported similar reductions, with the active phase shortened from 3.85 to 2.52 hours and the second stage from 65.5 to 48.1 minutes in the LCG group. Pandey et al. [17] also documented a nearly two-hour reduction in active labour duration with LCG use. Singh et al. [21] observed that 59% of women in the LCG group delivered within four hours compared to 39% in the Modified Partograph group. These findings suggest that the LCG enables timely clinical decision-making while avoiding premature intervention, thereby improving labour efficiency and maternal recovery.

The observed benefits of the WHO LCG in the present study are consistent with emerging global evidence supporting its adoption. Hofmeyr et al. [22] emphasized that the LCG overcomes the limitations of traditional partographs by eliminating rigid definitions of labour onset and allowing individualized monitoring. Ghulaxe et al. [23] highlighted the LCG’s modular structure, which facilitates continuous reassessment and timely intervention, while Patabendige et al. [24] described the LCG as a paradigm shift toward respectful and woman-centred intrapartum care. Lee et al. [25] demonstrated that revised monitoring formats improved detection of labour dystocia without increasing intervention rates. Together with the findings of the present study, this growing body of evidence supports the WHO LCG as a clinically effective, safe, and contemporary tool for labour monitoring, particularly when accompanied by adequate training and adherence to recommended protocols.

The strengths of the present study include its prospective comparative design, well-matched case and control groups, and strict adherence to standardized WHO-recommended intrapartum monitoring tools, which together enhance the reliability and internal validity of the findings. Baseline comparability with respect to key demographic and clinical variables minimized confounding and allowed meaningful assessment of the impact of the WHO LCG versus the WHO Modified Partograph on labour outcomes. Additionally, the study contributes valuable institution-based evidence from a low-resource setting, adding to the limited comparative literature on these two monitoring tools.

However, the study had a few limitations. The small sample size (n = 146) and single-centre nature of the study limit the generalizability of the results to other healthcare settings. Furthermore, qualitative aspects such as healthcare provider acceptability, ease of use, training requirements, and maternal satisfaction with the LCG were not assessed.

## Conclusions

The present study demonstrates that the WHO LCG is a superior intrapartum monitoring tool compared with the WHO Modified Partograph for primigravida women at term, offering significant improvements in maternal outcomes without compromising neonatal safety. The use of the LCG was associated with more efficient labour progression, reflected by shorter durations of the active and second stages of labour, a lower caesarean section rate, and reduced hospital stay, while neonatal outcomes remained comparable between groups. These findings support the LCG's capacity to facilitate timely, individualized clinical decision-making and align intrapartum care with contemporary, woman-centred obstetric practice. Despite limitations related to sample size and single-centre design, the results provide meaningful evidence in favour of integrating the WHO LCG into routine obstetric practice, with larger multicentric studies warranted to further validate its effectiveness and implementation feasibility.
